# Early Post-Operative Diagnosis of Anastomotic Leak by Local Electrophysiological Parameters: Multicenter Pilot Study

**DOI:** 10.3390/diagnostics16182969

**Published:** 2026-09-14

**Authors:** Elchanan Quint, Lior Segev, Vladimir Gaziants, Uri Netz, Avi Reshef, Ian White, Yulie Hagani, Udi Shenker, Gilad Lerman, Ilana Fishman, Shai Policker, Erez Shor, Hagit Tulchinsky, Ilia Pinsk, Rafael Miller, Barak Bar-Zakai, Oded Zmora, Nir Wasserberg, Matan Ben-David, Charles Knowles

**Affiliations:** 1 Soroka University Medical Center, Beer-Sheva 84101, Israel; elchananq@gmail.com (E.Q.); urinetz@gmail.com (U.N.); areshef1@yahoo.com (A.R.); iliapi@clalit.org.il (I.P.); 2 Faculty of Health Sciences, Ben-Gurion University of the Negev, Beer-Sheva 84105, Israel; 3 Sheba Medical Center (Tel HaShomer), Ramat Gan 52621, Israel; lior.segev@sheba.health.gov.il; 4 Gray School of Medicine, Tel Aviv University, Tel Aviv 69978, Israel; ian.white@clalit.org.il (I.W.); hagitt@tlvmc.gov.il (H.T.); zmorao@shamir.gov.il (O.Z.); nirw@clalit.org.il (N.W.); 5 Kaplan Medical Center, Rehovot 76100, Israel; vgaziants@gmail.com (V.G.); rafi_mi@clalit.org.il (R.M.); barakba@clalit.org.il (B.B.-Z.); 6 Faculty of Medicine, Hebrew University of Jerusalem, Jerusalem 91121, Israel; 7 Beilinson Hospital, Rabin Medical Center, Petah Tikva 49100, Israel; 8 Exero Medical, Jersey City, NJ 07305, USAshai@edgemed.vc (S.P.); erez.shor@exeromedical.com (E.S.); matan.bendavid@exeromedical.com (M.B.-D.); 9 Tel Aviv Medical Center, Tel Aviv 64239, Israel; 10 Shamir Medical Center (Assaf Harofeh), Beer Yaakov 70300, Israel; 11 Barts and The London School of Medicine & Dentistry, Queen Mary University of London, London E1 4NS, UK; 12 Cleveland Clinic London, London SW1X 7HY, UK

**Keywords:** anastomotic leak, early detection, post-op surveillance, continuous physiologic monitoring

## Abstract

**Background**: Morbidity associated with anastomotic leak (AL) can be reduced by timely detection. Local electrophysiological parameters are highly sensitive to pathophysiological processes (inflammation/ischemia) underlying AL and may enable early post-op monitoring ahead of clinical signs. A new approach to early leak detection includes post-operative measurement of bioelectric activity of the GI tract. The method has been implemented in a medical device that uses electrodes embedded in a standard surgical drain to continuously monitor local bowel myoelectric activity following GI surgery. **Methods**: A multicenter pilot study evaluated the performance of the device to support detection of clinically recognizable anastomotic leaks on post-operative day 3. The device’s technology collected data that an algorithm classified to predict AL. Data were collected for a duration of 3–10 days, while the surgical drain was in place (without affecting routine care). Pilot endpoints were safety and performance analyses: sensitivity and specificity. **Results**: Fifty patients from 6 medical centers (mean age: 63.6 ± 13.3 years; 38% male) undergoing elective anterior resection (laparoscopic (*N* = 26), robotic (*N* = 16), open (*N* = 8)) for cancer (*N* = 44), diverticulitis (*N* = 4), and rectal prolapse (*N* = 2) were included. Average anastomotic height was 9 ± 5 cm from the anal verge. Grade C ALs were observed in 6.0% (*N* = 3) of the patients, resulting in increased hospitalization time (6.4 ± 2.4 days with no leak vs. 15.0 ± 7.9 days with Grade C leak). In this *N* = 50 cohort, the device had 100% sensitivity and 100% specificity at POD3 in prediction of Grade C leaks. Given the small number of events (three Grade C leaks, seven leaks overall), these results are preliminary. No device-related serious adverse events were observed, and the standard drain form factor contributed to positive usability feedback. **Conclusions**: Pilot data suggest that the bioelectric sensing drain device is safe and warrants further evaluation as a potential adjunct for AL detection. The small study sample size and wide confidence intervals for key outcomes require confirmation in a pivotal diagnostic accuracy study with a fixed thresholding algorithm.

## 1. Introduction

Anastomotic leakage (AL) is one of the most serious complications following colorectal surgery, with significant mortality ranging from 7.5% to 39% [1,2,3]. Delay in diagnosis and intervention significantly worsens outcomes and increases the mortality rate [4,5]. Timely diagnosis of AL remains a clinical challenge. Current methods rely on signs and symptoms, systemic inflammatory markers, imaging, and clinical judgment. Symptoms are often nonspecific and may not present until sepsis is significant [6], and computed tomography has limited sensitivity in the early postoperative period [7,8]. Likewise, systemic biomarkers such as C-reactive protein (CRP) and WBC are non-specific and have low positive predictive value (21–23%) [6].

Growing evidence suggests that local tissue changes, including ischemia, inflammation, and edema, precede systemic manifestations of AL. These changes alter the bioelectrical properties of the bowel [9] and gastrointestinal (GI) myoelectric activity, offering a potential window for early detection [10,11,12]. Inflammation and ischemia [13] have been shown to affect impedance, electrical slow-wave activity, and spike generation in the gut, correlating with altered motility, prior to systemic signs and symptoms.

We have previously shown in ex vivo human tissue that acute ischemia caused a rapid and measurable reduction in the spike rate and power of myoelectric activity near the anastomosis in patients undergoing colorectal surgery [14]. These findings suggest the potential use of real-time electrophysiological monitoring to assess tissue viability and potentially detect AL before clinical signs emerge. In addition, changes in tissue impedance measured by electrodes located at the surgical site detect local inflammation associated with AL in animal models [15]. Together, these studies suggest that continuous sensing of mucosal electrophysiology and tissue impedance may offer a novel approach for early detection of AL.

The xBar system (Exero Medical, Israel) is an implantable device designed to continuously measure both myoelectric activity and impedance near the anastomosis. By comparing readings at the anastomotic site to a proximal reference point, the system detects local deviations that may indicate early anastomotic compromise. A prior feasibility study confirmed the device’s safety and operability in the postoperative setting [14].

This prospective multicenter pilot study evaluated the safety and functionality and estimated the utility of this device system as potential adjunctive decision support for early detection of colorectal AL through real-time, local physiologic monitoring.

## 2. Materials and Methods

### 2.1. Study Design and Setting

This was a prospective, multicenter, observational study designed to provide pilot data relevant to the safety, feasibility, and detection performance of the xBar system for early detection of colorectal AL. The study was conducted at 6 tertiary hospitals between March 2022 and January 2023, following institutional review board (IRB) approval at each site. Written informed consent was obtained from all participants prior to enrollment.

### 2.2. Participants

Eligible patients were ≥22 years old and scheduled to undergo elective anterior resection with a colorectal or coloanal anastomosis. Exclusion criteria included: known allergy to implant materials, pregnancy, electronic device implanted in the chest or abdominal cavity, participation in another investigational trial during the system usage, exposure to MRI during the system usage.

### 2.3. Device Description

**Figure 1:**
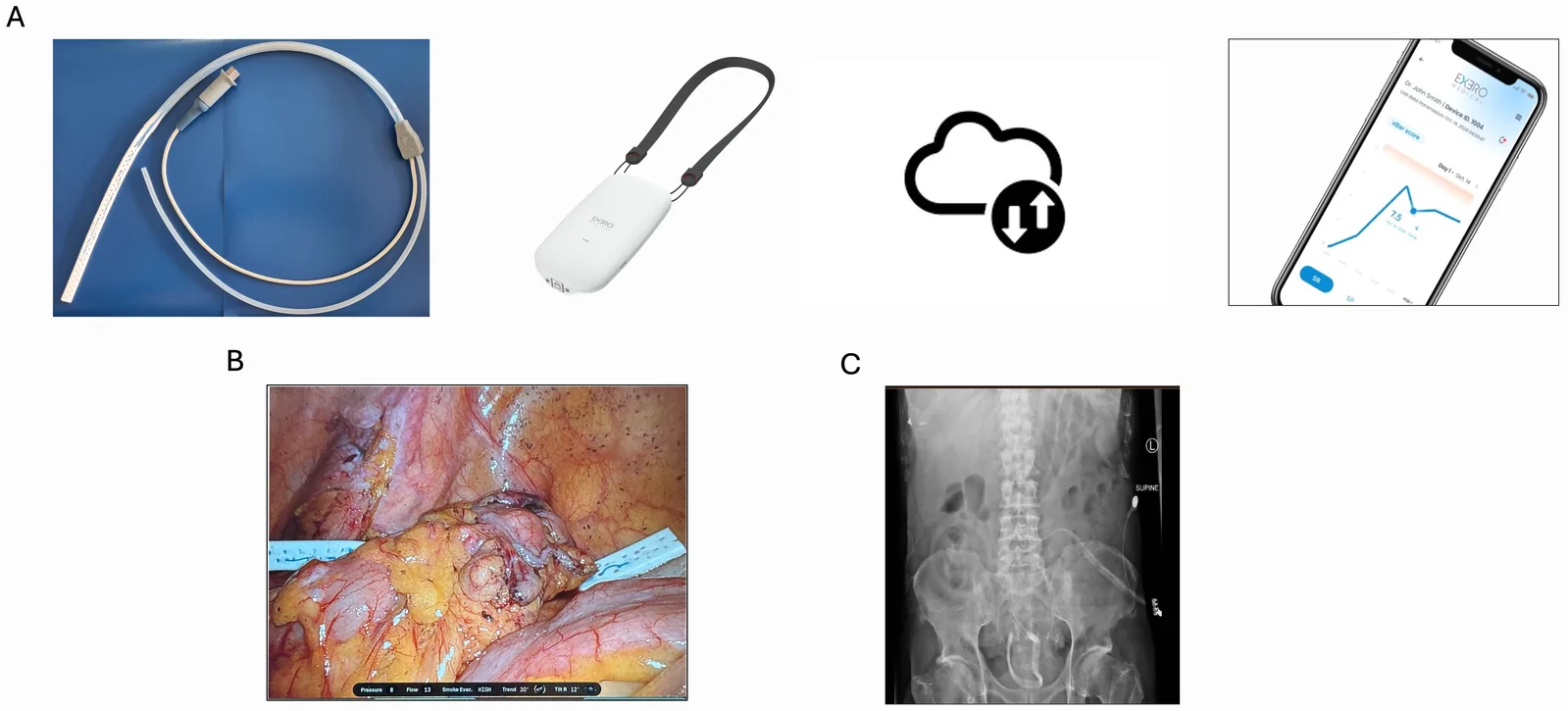
Overview of the xBar system. (**A**) The electrode array is embedded into a standard surgical drain. The drain is connected to a standard fluid reservoir as well as to an electronic device, which records myoelectric data and relays it to a cloud database where data is stored and processed. The clinical team receives indications on a web app. The system can be used in open, laparoscopic, and robotic surgeries. (**B**) Intraoperative image of xBar placement, which is similar to any drain placement; no active alignment is required. (**C**) Plain radiograph showing drain placement.

xBar is an investigational implantable single-use sensor integrated within a standard surgical drain (10 mm Jackson–Pratt). An array of electrodes (typically 1 mm long) embedded on the surface of the drain continuously records electrical impedance and myoelectric activity from tissues adjacent to the anastomosis. The design of the electrode placement enables simultaneous recording from sites close and further away from the anastomosis [14]. Data are recorded by a small wearable device and stored for analysis. The system is described in Figure 1.

### 2.4. Surgical Procedure and Device Implantation

At the final stage of the surgical procedure (whether robotic, laparoscopic, or open) and prior to abdominal cavity closure, the xBar drain was positioned in the abdominal/pelvic cavity as per routine surgical practice. Device positioning was confirmed visually by the operating surgeon. At the end of the surgery, the xBar drain was connected to an electronic device recording and storing the data.

### 2.5. Data Collection and Follow-Up

Continuous data acquisition began intraoperatively and continued for up to 7 postoperative days or until drain removal. Device performance and safety were monitored daily. Routine clinical data, including CRP, operative notes, and postoperative follow-up notes, were collected. Diagnosis and detection of AL were made based on clinical, radiological, and/or intraoperative findings, in accordance with institutional guidelines and international classification to grades A–C. Protocol pilot endpoints were safety (absence of device-related serious adverse events) and functional success (successful implantation followed by recording of data for 4 consecutive days or more). xBar recorded raw data from a set of 8 electrodes using an ADS1299 analog-to-digital converter (Texas Instruments, Dallas, TX, USA). Raw data were acquired at 250 Hz.

### 2.6. Signal Processing and Analysis

**Figure 2:**
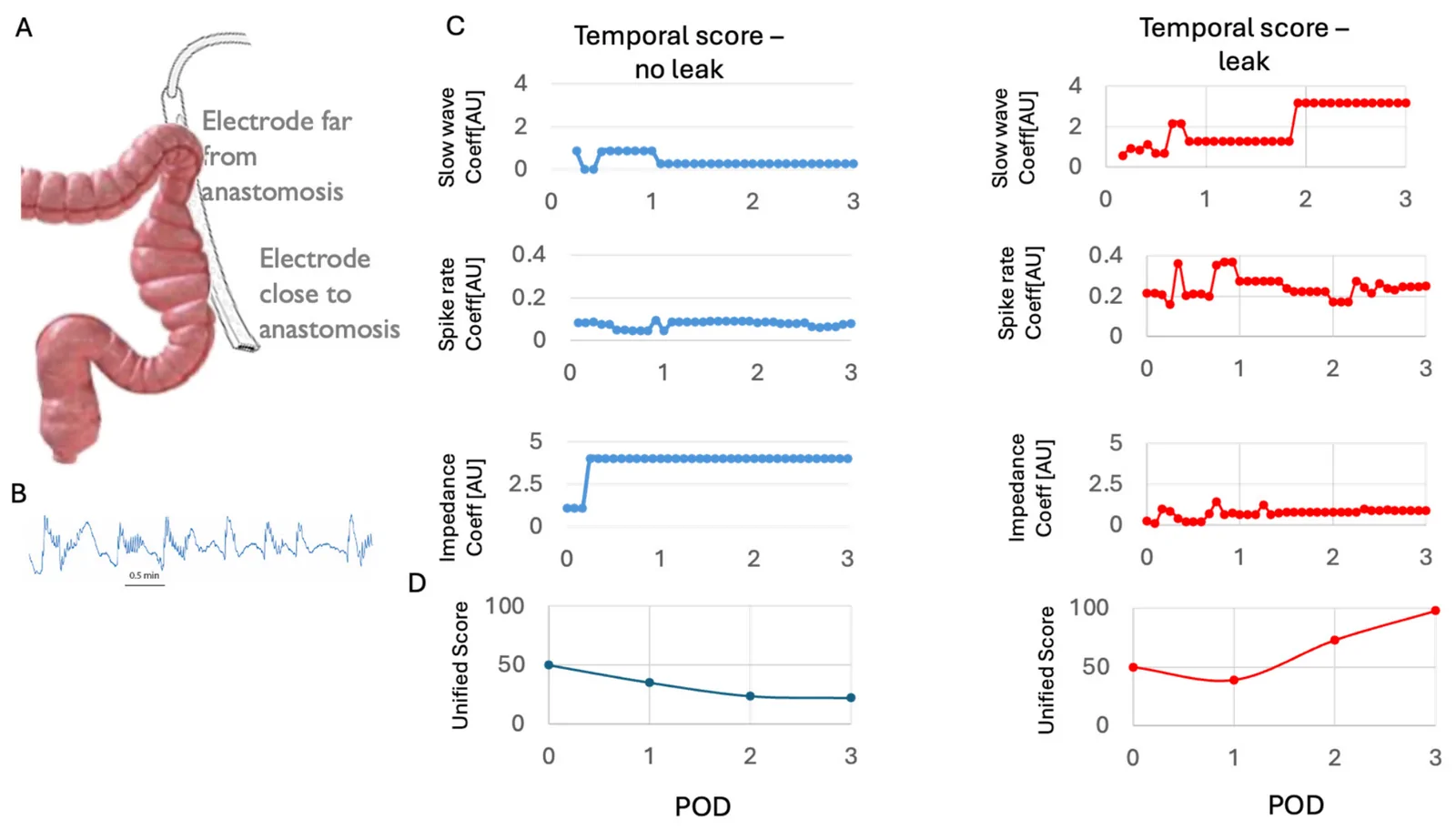
xBar signal characterization. (**A**) The drain records information from multiple electrodes at varying distances to the anastomosis. Impedance and myoelectric signals are collected. (**B**) Typical raw signal trace showing slow-wave oscillations with higher-frequency spikes. (**C**) The system calculates temporal signals driven by the slow waves, spike rate, and impedance, and expresses them graphically as coefficients. Low slow-wave and spike coefficients are indicative of relatively small differences between electrodes near the anastomosis and farther away from it. Differences in the trajectory and variability, alongside low impedance measurements, are observed in a typical patient with a Grade C anastomotic leak. (**D**) A unified score derived from all 3 variables can be calculated for each patient. In those with anastomotic leak, the score rises over time in contrast to the falling trajectory in a patient without a leak.

Data were acquired for three groups of signals: impedance, rate of slow waves (f < 0.2 Hz), and higher frequency content (1 < f < 125 Hz) associated with the contractility of the colon muscle (spikes, defined as instances where the signal crossed a 2-sigma threshold for a short period of time) (Figure 2). The impedance coefficient is a descriptive function of the temporal impedance change trends across the electrodes. Inconsistent trend changes across electrodes (being placed near and farther away from the anastomosis) are indicative of different tissue characteristics near and farther away from the anastomosis. Similarly, spike rate and slow-wave coefficients are the differences between spiking activity and slow waves, respectively, near and farther away from the anastomosis. They are indicative of different colonic contraction force and pacing near the anastomosis and farther away from it. Therefore, low slow-wave and spike coefficients stem from relatively small differences between electrodes near the anastomosis and farther away from it, which is indicative of similar function of the area near the anastomosis and remote, intact colonic tissue.

For each coefficient, a score was derived by normalizing the temporal coefficient values to score values between 0 and 1, where 0 indicates no difference between the electrodes near and the anastomosis and farther away from it, and 1 was taken as the maximum coefficient observed in the study. Multiplication of the 3 scores could derive a unified score for each patient (see below). Scores were evaluated at POD1, 2, and 3 and compared with the actual diagnosis of the clinical team over time, who were blinded to the xBar data.

Handling of missing data: Missing data were not imputed. The system incorporates spatial and temporal redundancy to handle data loss at the source: if a single electrode’s signal was unavailable, spatial redundancy from the remaining electrodes was used to substitute the missing value (mean or median across electrodes). If a temporary device disconnection caused a data outage, the missing recording was substituted using the temporal mean or median calculated over the preceding 6 h recording window. There were no cases with outages >12 h. Although the protocol required 4 days of recording, the analysis used data from the first three post-operative days only.

Missing CRP data: CRP values were not available for all patients on all postoperative days (e.g., due to blood draws not performed per local clinical practice); no imputation was performed for missing CRP values, and analyses were conducted using all available observations (complete-case basis). Missingness was confined to the no-leak group: CRP was available for 36/43, 34/43, and 37/43 no-leak patients at POD1, POD2, and POD3, respectively, while all patients with a Grade B or Grade C leak had complete CRP data at every POD (43/50, 41/50, and 44/50 patients overall at POD1–3).

### 2.7. Statistical Analysis

Descriptive statistics were used to summarize demographic and clinical data. Continuous variables are presented as means ± standard deviation or medians with interquartile ranges, as appropriate. Categorical variables are presented as counts and percentages. A memoryless decision rule classifier with fixed thresholds was trained on three features. Discriminative performance, independent of any threshold, was summarized as the area under the receiver operating characteristic curve (AUC), calculated across the full pilot cohort (*n* = 50) for each postoperative day (POD) and leak grade. A single detection threshold was derived from POD3 non-leak values as the maximum observed value plus a 10% margin, and applied without modification across all PODs and leak grades (Grade C, Grade B + C). Threshold stability was assessed using leave-one-out (LOO) cross-validation at POD3: the threshold was re-derived after excluding each patient in turn, and that patient’s classification was based on the re-derived threshold. Sensitivity, specificity, PPV, and NPV are reported with 95% confidence intervals calculated using the Clopper-Pearson exact method. With only 3 Grade C and 4 Grade B leak events in the pilot cohort, this threshold should be regarded as a candidate operating point requiring confirmation, with pre-specification, in an adequately powered study.

Limited statistical comparison was made for the purpose of data presentation—in keeping with pilot design. To compare means between two independent groups, an unpaired two-tailed Student’s *t*-test was used. We used linear mixed-effects models (LMM) with leak grade, postoperative day (POD), and their interaction as fixed effects, and a patient-level random intercept to account for repeated measures. Uniscore was log-transformed prior to modeling given its several-orders-of-magnitude dynamic range and right-skewed distribution; CRP was modeled on its raw scale. Fixed-effect significance was assessed via Wald chi-square tests, and the Grade × POD interaction was additionally evaluated by a likelihood-ratio test comparing models with and without the interaction term (fit by maximum likelihood). Intraclass correlation coefficients (ICC) were calculated to quantify the proportion of variance attributable to between-patient differences. Because residual diagnostics indicated persistent non-normality, we performed a sensitivity analysis using the Wilcoxon rank-sum (Mann–Whitney U) test, a distribution-free method robust to outliers and non-normality, comparing Grade C and Grade B against the no-leak group independently at each POD. All tests were two-sided except where a directional hypothesis (Uniscore/CRP elevation in leak groups) justified a one-sided test, as noted. Significance was set at α = 0.05; where multiple pairwise comparisons were performed, Bonferroni correction was applied. Statistical significance was set at *p* < 0.05. All analyses were performed using Python ver. 3.10.

### 2.8. Clinical Diagnosis and Classification of Leaks

Leaks were classified according to the International Study Group of Rectal Cancer [16] as Grade B (bedside intervention) and Grade C (surgical re-intervention). Postoperative X-ray was mandatory for all patients to confirm device placement, not leak diagnosis; CT was performed when a leak was clinically suspected. Grade C leaks were confirmed by contrast CT and at re-intervention; Grade B by CT alone. For the purposes of this study, the target condition (anastomotic leak, AL) refers to clinically recognized leakage requiring intervention (Grade B or Grade C); this is distinct from anastomotic leak in the broader sense, which may include radiologically apparent but clinically silent (Grade A) leaks. Patients without clinical suspicion did not undergo CT and were classified as no leak, which may include undetected subclinical (Grade A) leaks [17]. The clinical team was blinded to xBar output throughout.

### 2.9. Role of the Sponsor

The study was sponsored by Exero Medical Ltd. Exero scientific staff participated in ensuring the correct deployment of the xBar device and analysis of data. However, signal processing was undertaken blind to the clinical status of the patient. xBar output, including threshold derivation and classification, was generated retrospectively after study completion. The detection threshold was derived using outcome data from the full cohort and was not available in real time; treating clinicians remained blinded to xBar output throughout the study, and the device did not generate a real-time alert or influence clinical decision-making at any point.

## 3. Results

### 3.1. Patient Characteristics

A total of 50 patients were enrolled in the study. The mean age was 63.6 ± 13.3 years, and 62% were female. Patient demographics and clinical characteristics are summarized in Table 1.

Neoadjuvant therapy was administered to a subset of patients, including chemotherapy alone, radiotherapy alone, and combined chemoradiotherapy. The primary indications for surgery included colorectal cancer, rectal prolapse, and diverticular disease.

Surgical approaches varied across the cohort: laparoscopic surgery was performed in 26 patients (52%), robotic surgery in 16 patients (32%), and open surgery in 8 patients (16%). The mean distance from the anastomosis to the anal verge was 9.1 ± 5.3 cm. The rate of diverting ileostomy was 44% (*N* = 22). Anastomoses were performed using stapling devices in all patients.

### 3.2. Postoperative Outcomes

Among the 50 patients, 7 (14%) experienced anastomotic leakage: 4 patients were classified as Grade B leaks (requiring bedside intervention), all of whom were diverted, and 3 as Grade C leaks (requiring surgical re-intervention), none of whom were diverted. All leaks were clinically confirmed using CT. Three patients presented additional serious adverse events without evidence of a leak, and 3 had minor complications unrelated to the anastomosis.

The mean length of hospitalization varied markedly across groups. Patients without complications had an average hospital stay of 6.2 ± 2.0 days (range: 3–13), compared to 10.8 ± 2.2 days for Grade B leaks, and 15.0 ± 7.9 days for Grade C leaks. Patients with other major complications had a similarly prolonged hospitalization (17.6 ± 10.0 days, range: 5–28). Patients were followed postoperatively for up to 100 days after the index surgery. Postoperative outcomes are summarized in Table 2.

**Table 2:** Postoperative complications and length of hospital stay.

Group	*N*	Hospitalization Days (Mean ± SD)	Range (Days)
No Severe Complications	38	6.42 ± 2.42	3–14
Anastomotic Leak—Grade B	4	10.75 ± 2.22	9–14
Anastomotic Leak—Grade C	3	15.0 ± 7.94	6–21
Major Complication (No Leak Detected)	5	17.60 ± 9.96	5–28

### 3.3. xBar Signal Analysis and Leak Detection

xBar successfully recorded and processed localized electrical signals in 50 patients. From the 50 patients enrolled, two patients withdrew consent on the third and fourth day postoperatively. However, both patients recorded sufficient data to support sensitivity and specificity analysis at POD 2–3.

Signal monitoring (Figure 2) produced raw signal traces showing slow-wave oscillations with higher-frequency spikes, from which temporal traces could be derived and expressed graphically as coefficients. Differences in the trajectory and variability in slow-wave and spike activity varied between patients with and without leak. Impedances also changed over time, with persistently low impedances typically found in subjects with anastomotic leak. The unified score (uniscore) was derived from all three variables for all patients. In those with anastomotic leak, the score typically rose over time in contrast to falling trajectories in patients without a leak.

**Figure 3:**
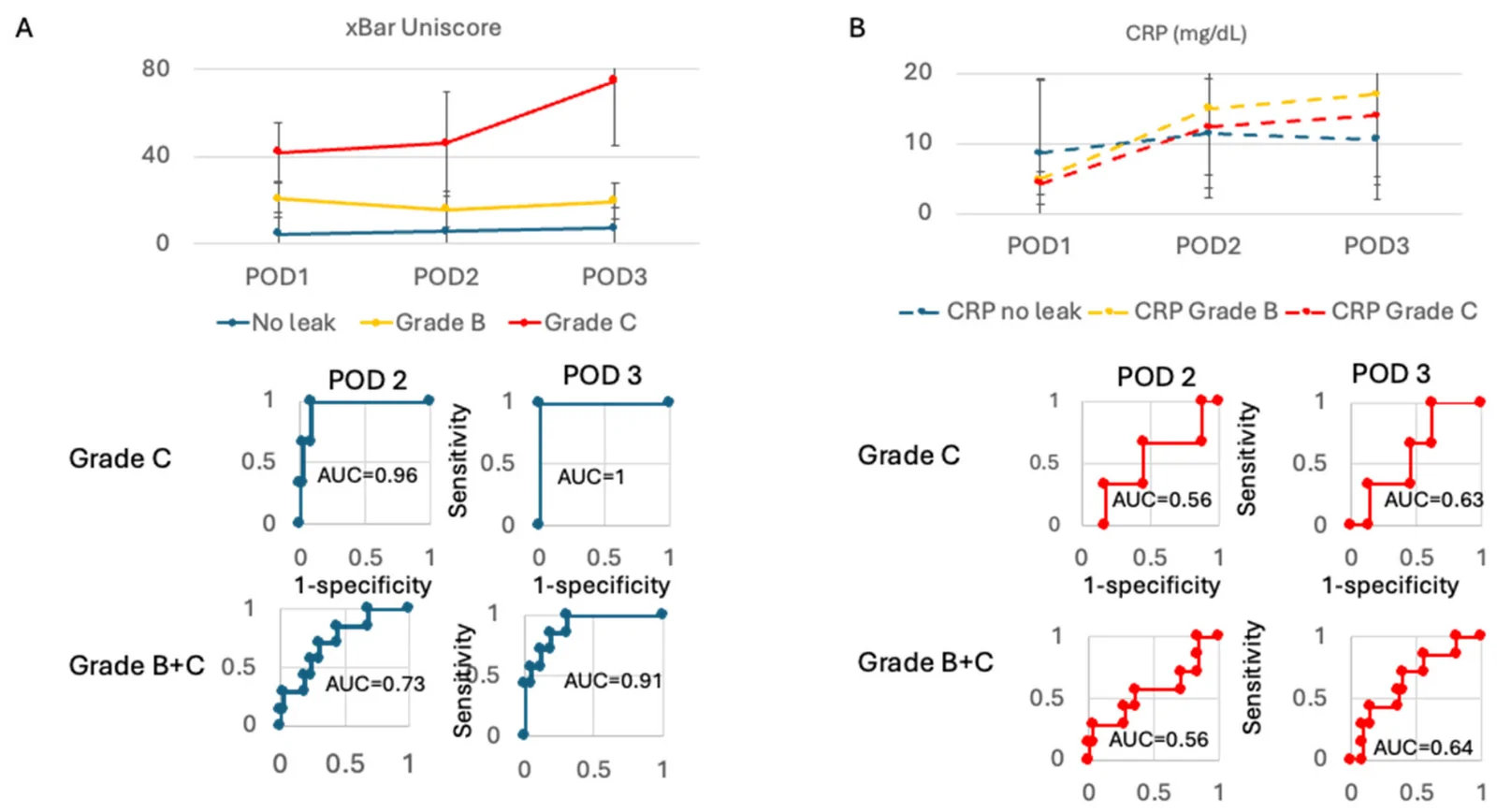
Leak detection results. (**A**) The Xbar device’s unified score is shown for no leak (*N* = 43), Grade B leak (*N* = 4), and Grade C leak (*N* = 3). Error bars are SD. ROC for leak detection at POD 2 and 3 are shown for Grade C leaks and all leaks. (**B**) Comparison to CRP, which is the standard of care at POD 2-3, with its corresponding detection ROCs.

To assess its ability to detect anastomotic leaks, the xBar system generated a unified score (Uniscore) derived from spike rate, slow-wave activity, and impedance values, computed daily for each patient. Uniscore derivation, threshold calculation, and validation are described in the methods section. Figure 3A shows the progression of the Uniscore over postoperative days (POD) 1 to 3, stratified by outcome: no leak (*n* = 43), Grade B leak (*n* = 4), and Grade C leak (*n* = 3) (Figure 3A). Uniscore values were higher in patients with Grade C leaks compared to those with no leak or Grade B leaks across POD1–3, reaching 74.6 ± 29.5 in Grade C by POD3 versus 6.9 ± 9.9 in non-leak and 19.4 ± 8.2 in Grade B patients. Accounting for within-patient correlation (ICC = 0.86) using a linear mixed-effects model on log-transformed Uniscore, leak grade showed a significant main effect (χ^2^ = 7.64, df = 2, *p* = 0.022) and POD was highly significant (χ^2^ = 58.75, df = 2, *p* < 0.001); the grade × POD interaction was not significant (likelihood-ratio test, χ^2^ = 4.40, df = 4, *p* = 0.355). Given the extreme dynamic range and non-normality of Uniscore, we additionally performed a Wilcoxon rank-sum sensitivity analysis, which showed a separation between Grade C and no-leak patients at POD2 (*p* = 0.0007) and POD3 (*p* = 0.0001). This indicates that Uniscore differed in overall level between leak grades and increased over time across the cohort as a whole, but does not provide statistical evidence that the rate or shape of change over time differed between leak grades. Given the small Grade C sample size (*n* = 3), these comparisons should be interpreted as descriptive/hypothesis-generating rather than confirmatory.

By comparison, On POD3, mean CRP values were 14.0 ± 9.9 mg/dL in Grade C, 17.0 ± 11.8 mg/dL in Grade B, and 10.5 ± 8.4 mg/dL in non-leak patients (Figure 3B). A linear mixed-effects model accounting for within-patient correlation (ICC = 0.54) showed no significant main effect of leak grade (χ^2^ = 0.96, df = 2, *p* = 0.62), a borderline effect of POD (χ^2^ = 5.19, df = 2, *p* = 0.075), and no significant grade × POD interaction (χ^2^ = 7.09, df = 4, *p* = 0.13). Wilcoxon rank-sum tests comparing each leak grade against the no-leak group at each POD were likewise non-significant throughout (all *p* ≥ 0.16), corroborating the limited utility of CRP for distinguishing leak severity in the early postoperative period.

The Uniscore discriminated Grade C leaks from all other patients with an AUC of 1.0 at POD3 and 0.96 at POD2 (full pilot cohort). Discrimination of the combined Grade B + C group from no-leak patients was more modest, with an AUC of 0.9 at POD3 and 0.7 at POD2.

A single detection threshold was derived from POD3 non-leak values and applied without modification across all PODs and leak grades. Applying this fixed threshold yielded the following results:

• POD3, Grade C: sensitivity 100% (3/3, 95% CI 29.2–100), specificity 100% (47/47, 95% CI 92.5–100), PPV 100% (3/3, 95% CI 29.2–100), NPV 100% (47/47, 95% CI 92.5–100).
• POD2, Grade C: sensitivity 66.7% (2/3, 95% CI 9.4–99.2), specificity 97.9% (46/47, 95% CI 88.7–99.9), PPV 66.7% (2/3, 95% CI 9.4–99.2), NPV 97.9% (46/47, 95% CI 88.7–99.9).
• POD3, Grade B + C: sensitivity 42.9% (3/7, 95% CI 9.9–81.6), specificity 100% (43/43, 95% CI 91.8–100), PPV 100% (3/3, 95% CI 29.2–100), NPV 91.5% (43/47, 95% CI 79.6–97.6).
• POD2, Grade B + C: sensitivity 28.6% (2/7, 95% CI 3.7–71.0), specificity 97.7% (42/43, 95% CI 87.7–99.9), PPV 66.7% (2/3, 95% CI 9.4–99.2), NPV 89.4% (42/47, 95% CI 76.9–96.5).

In comparison, C-reactive protein (CRP) levels (Figure 3B) showed overlapping trajectories between no-leak and leak groups on PODs 2 and 3. CRP ROC analysis revealed limited discriminative performance, with AUCs ranging from 0.56 to 0.64 across POD 2 and 3 for both Grade C and Grade B + C leak detection (Figure 3B). CRP ROC analyses were performed on all patients with an available CRP value at the corresponding POD; all leak patients (Grade B and C) had complete CRP data, so this reflects missingness in the no-leak group only, not differential missingness between leak and no-leak patients.

### 3.4. Detection Time

**Figure 4:**
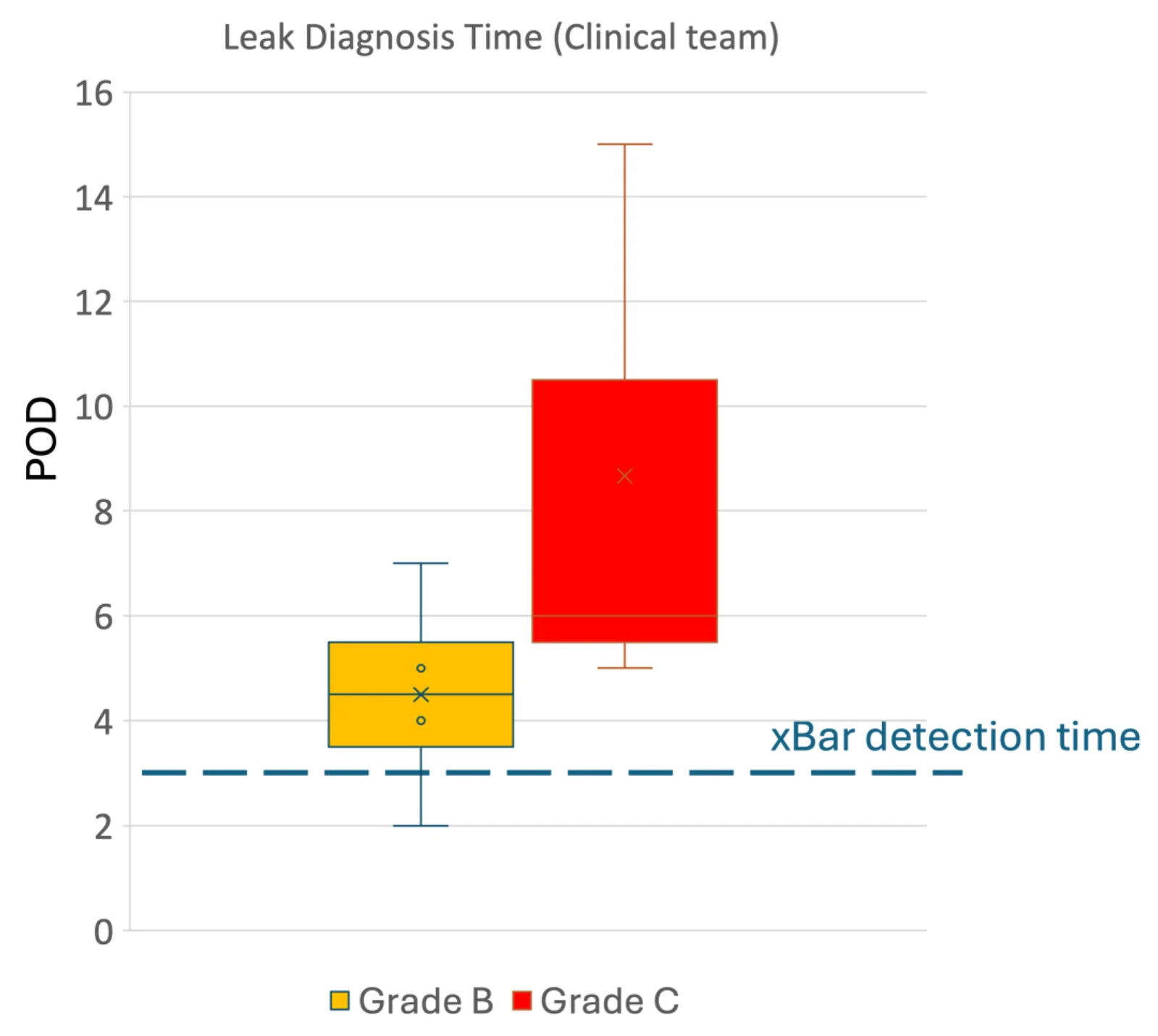
Time to leak diagnosis in Grade B and Grade C leaks compared to xBar’s detection time.

Leak detection time by the clinical team was 6.3 ± 4.2 days (*N* = 7, with 8.7 ± 5.5 days for Grade C leaks, *N* = 3 and 4.5 ± 4.0 days for Grade B leaks *N* = 4). As shown in Figure 4, retrospective analysis of xBar signals indicated leak likelihood by POD3.

### 3.5. Usability

Following each surgery, the surgeons completed a usability questionnaire where they were asked to score xBar’s use, training time, and overall satisfaction on a scale ranging from 1 (low score) to 5 (high score). The results in Table 3 indicate surgeons were pleased with xBar’s usability.

## 4. Discussion

Anastomotic leakage remains one of the most devastating complications of colorectal surgery, with significant consequences for patient outcomes and healthcare burden. Impedance-based detection of leaks has been attempted [18] but failed to translate to the clinic. Emerging intra-op leak prevention modalities such as ICG still fail to provide a significant measure to prevent anastomotic leaks [19,20].

In this multicenter pilot study, we demonstrate that continuous monitoring of local physiological signals post-op using xBar enabled, on retrospective analysis, at least in the population studied, early indication of patients eventually developing a clinically recognized colorectal anastomotic leak requiring intervention. Such data could be used to derive a score based on three measured variables: myoelectric spike activity, slow-wave activity, and impedance, with cut-offs that enabled high levels of discrimination when applied to this population. The form factor of a standard drain was easy to use and was successfully deployed in all 50 patients, with no device-related adverse events, highlighting its feasibility and safety.

With the caveat of the pilot design and absence of an independent test sample, the system showed promising accuracy for detection of clinically confirmed leaks with high observed sensitivity and specificity at POD 3 compared to CRP, the most widely used biomarker in current practice. This study does not propose a head-to-head diagnostic accuracy comparison of two equivalent tests: CRP is a systemic marker and therefore subject to noise from inflammatory processes other than anastomotic leaks, limiting its specificity. xBar is designed to collect data local to the anastomosis itself. This proposes a mechanistic rationale for xBar’s role as an adjunct to systemic markers such as CRP.

Notably, diversion status was confounded with leak grade in this cohort (Grade B leaks occurring exclusively in diverted patients, Grade C exclusively in non-diverted patients). Diversion alters intestinal continuity, luminal contents, local pressure, and the regional inflammatory response, any of which could independently affect the measured electrophysiological signal. It is plausible that weaker xBar signal separation for Grade B reflects lower leak severity, a diversion-related dampening of the physiological signal, or both. While this study provides preliminary evidence for discrimination of clinically confirmed leaks, particularly Grade C leaks, the relationship between the electrophysiological signal, diversion status, and leak severity remains unresolved. However, these results confirm prior preclinical observations [15] and intraoperative findings [14] suggesting that early local pathophysiology precedes systemic markers of severe leaks and is measurable using bioelectrical signals.

Given the well-established correlation between delayed intervention and increased mortality in AL, it is plausible that leak likelihood indication by xBar’s at POD3, supported by additional diagnostics, may lead to a potential reduction in the clinical detection time. Acceleration in leak detection could guide imaging and triage to reoperation or drainage.

Several key limitations are implicit with the pilot study design and must be acknowledged. The sample size of 50, while among the largest for device-based leak monitoring to date, is insufficient to provide statistical power for key findings. Further, with only seven leaks, it was clearly insufficient to report any observations on device performance in subgroups such as patients with or without neoadjuvant therapy, proximal diversion, or types of anastomosis. The next major limitation concerns device performance being based on training within the dataset. While the POD3 Grade C result was robust to leave-one-out perturbation, this constitutes internal validation within the derivation cohort, not external validation in an independent population. A pre-specified threshold, fixed prior to data collection, should be evaluated in an adequately powered, multicenter pivotal study before diagnostic performance claims are made for clinical use. We also acknowledge the study participation of the commercial sponsor. This was necessary to ensure correct clinical implementation of a new technology and is acceptable for a pilot investigation. We, however, acknowledge that this may introduce bias in the analysis and inference from data, but note that all signal processing was undertaken blinded to clinical outcomes. Finally, clinical diagnosis of leaks was made per standard of care and not adjudicated centrally.

Future pivotal studies with predefined thresholds, adjudicated endpoints, and integration with clinical workflow will be needed to validate these findings in a broader context. In the future, a prospective interventional study will hopefully demonstrate significant impact on the clinical course patients undergo following gastrointestinal surgery.

## 5. Conclusions

In conclusion, xBar represents a promising new modality enabling earlier detection of clinically recognized anastomotic leaks requiring timely intervention. In this pilot cohort, the device generated a signal before conventional clinical diagnosis and could be safely and seamlessly integrated into the surgical workflow. The clinical utility and impact on patient management remain to be established prospectively, and an adequately powered prospective study is warranted before clinical implementation can be recommended.

## Figures and Tables

**Table 1:** Baseline characteristics of the study cohort (*N* = 50).

Variable	Value
Gender, *n* (%)	Male: 19 (38%); Female: 31 (62%)
Age, mean ± SD (years)	63.6 ± 13.3
BMI	25.95 ± 4.34
Surgical Approach, *n* (%)	Open: 8 (16%); Laparoscopic: 26 (52%); Robotic: 16 (32%)
Anastomosis Distance, cm	9.1 ± 5.3 from anal verge
Neoadjuvant Therapy	14
Surgical Indication	Malignancy 43 (86%), Rectal Prolapse 1 (2%), Diverticulitis 3 (6%), other 3 (6%)
Ileostomy Created	22 (44%)

**Table 3:** Usability scores.

Usability Question	Average Score	Stdev	*N*
How would you rate the ease of use of deploying the SmartDrain (xBar) in the abdominal cavity?	4.57	0.63	42
How satisfied are you with the time it takes to learn how to use the SmartDrain (xBar) (i.e., learning curve)?	4.65	0.53	43
Overall, how would you rate the use of the SmartDrain (xBar)?	4.44	0.67	43

## Data Availability

The datasets generated and analyzed during the current study are proprietary property of Exero Medical and are not publicly available due to commercial confidentiality. De-identified or aggregated data may be made available from the corresponding author upon reasonable request.

## Abbreviations

The following abbreviations are used in this manuscript:

AL	Anastomotic Leak
CRP	C-Reactive Protein
GI	Gastro-Intenstinal
PPV	Positive Predictive Value
